# p53 controls the plasticity of mammary luminal progenitor cells downstream of Met signaling

**DOI:** 10.1186/s13058-019-1101-8

**Published:** 2019-01-25

**Authors:** Aurélie Chiche, Amandine Di-Cicco, Laura Sesma-Sanz, Laura Bresson, Pierre de la Grange, Marina A. Glukhova, Marisa M. Faraldo, Marie-Ange Deugnier

**Affiliations:** 1 Institut Curie, PSL Research University, CNRS, UMR144, 26 rue d’Ulm, F-75005 Paris, France; 2 Sorbonne Universités, UPMC Paris 06, F-75005 Paris, France; 3 Institut Pasteur, CNRS, UMR3738, F-75015 Paris, France; 4 Université Paris VII Denis Diderot, F-75013 Paris, France; 5 GenoSplice technology, F-75005 Paris, France; 6 INSERM, F-75013 Paris, France

**Keywords:** Mammary gland, Breast cancer, Stem cells, p53, Met

## Abstract

**Background:**

The adult mammary epithelium is composed of basal and luminal cells. The luminal lineage comprises two major cell populations, positive and negative for estrogen and progesterone receptors (ER and PR, respectively), both containing clonogenic progenitor cells. Deregulated ER/PR^−^ luminal progenitor cells are suspected to be at the origin of basal-type triple-negative (TNBC) breast cancers, a subtype frequently associated with loss of P53 function and MET signaling hyperactivation. Using mouse models, we recently reported that p53 restricts luminal progenitor cell amplification whereas paracrine Met activation stimulates their growth and favors a luminal-to-basal switch. Here, we analyzed how these two critical pathways interact to control luminal progenitor function.

**Methods:**

We have (i) established and analyzed the gene expression profile of luminal progenitors isolated by ICAM-1, a robust surface marker we previously identified; (ii) purified luminal progenitors from p53-deficient and p53-proficient mouse mammary epithelium to compare their functional and molecular characteristics; and (iii) analyzed their response to HGF, the major Met ligand, in three-dimensional cultures.

**Results:**

We found that luminal progenitors, compared to non-clonogenic luminal cells, overexpress *Trp53* and numerous p53 target genes. In vivo, loss of *Trp53* induced the expansion of luminal progenitors, affecting expression of several important p53 target genes including those encoding negative regulators of cell cycle progression. Consistently, p53-deficient luminal progenitors displayed increased proliferative and self-renewal activities in culture. However, they did not exhibit perturbed expression of luminal-specific markers and major regulators, such as *Hey1*, *Elf5*, and *Gata3*. Moreover, although expressing *Met* at higher level than p53-proficient luminal progenitors, p53-deficient luminal progenitors failed to acquire basal-specific features when stimulated by HGF, showing that p53 promotes the plastic behavior of luminal progenitors downstream of Met activation.

**Conclusions:**

Our study reveals a crosstalk between Met- and p53-mediated signaling pathways in the regulation of luminal progenitor function. In particular, it shows that neither p53 loss alone nor p53 loss combined with Met signaling activation caused an early detectable cell fate alteration in luminal progenitors. Conceivably, additional events are required to confer basal-specific characteristics to luminal-derived TNBCs.

**Electronic supplementary material:**

The online version of this article (10.1186/s13058-019-1101-8) contains supplementary material, which is available to authorized users.

## Background

Most of mammary gland development occurs postnatally under the control of female reproductive hormones. It is characterized by the establishment of a ductal tree during puberty and the formation of secretory alveoli at each pregnancy [1]. The postnatal mammary epithelium is organized as a bilayer, with a basal layer of myoepithelial cells and a luminal epithelial layer, largely maintained by distinct lineage-restricted stem cells or long-lived progenitors [2–4]. The luminal lineage, characterized by its expression of keratins 8/18/19, comprises ductal hormone-sensing cells, positive for estrogen and progesterone receptors (ER, PR), and ductal and alveolar cells lacking ER and PR expression [1, 5, 6]. Both ER/PR+ and ER/PR− luminal cell fractions contain clonogenic stem/progenitor cells that drive the expansion of the luminal cell population during puberty and gestation and ensure its maintenance at homeostasis [7–11].

The vast majority of breast cancers arise from deregulated luminal cells. Notably, ER/PR− luminal stem/progenitor cells are suspected to be at the origin of basal-like, triple-negative breast cancers (TNBCs), a subtype lacking ER, PR, and amplified HER2, displaying a basal-type gene signature [12–16]. Luminal progenitors seem therefore able to express basal-specific genes under pathological conditions, indicating that they display phenotypic plasticity, a property contributing to the complex intra-tumoral heterogeneity of TNBCs [17].

Several regulators of the mammary luminal cell lineage have been identified over the past years. We and other groups recently reported that luminal progenitors are targets of Met signaling [8, 18]. Paracrine activation of the Met tyrosine kinase receptor by its main ligand, the hepatocyte growth factor (HGF), stimulates the clonogenic activity of isolated luminal progenitors. In addition, it induces a luminal-to-basal switch, characterized by expression of basal-specific markers, induction of a partial epithelial-mesenchymal transition (EMT) program and acquisition of regenerative properties, a hallmark of bipotent basal stem cells [8].

The Met pathway controls multiple epithelial cell functions during development and tumorigenesis by regulating several signaling axes, including that of the tumor suppressor, p53 [19, 20]. Interestingly, p53 recently emerged as an important regulator of tissue development and homeostasis, controlling stem cell self-renewal and differentiation processes [21, 22]. Accordingly, using a mouse model with an ablation of p53 in the mammary epithelium, we found that loss of p53 resulted in the expansion of both basal and luminal compartments associated to a long-term amplification of their respective stem/progenitor cell populations [23].

Both aberrant MET activation and loss of P53 function are particularly frequent in TNBCs [24–27]. Met activation has been reported to promote p53 degradation in mouse epithelial cells [28], whereas loss of p53 can induce Met signaling activation [29, 30]. Moreover, recent studies suggest a synergy between p53 loss and Met activation in the formation of triple-negative mammary tumors in mouse models [31, 32].

Collectively, these data indicate that Met and p53 signaling pathways could crosstalk to control the function and plasticity of mammary luminal progenitors*.* To address this question, we isolated luminal progenitors from p53-deficient and p53-proficient mouse mammary epithelium, analyzed their molecular characteristics, and compared their response to HGF stimulation.

## Methods

### Mouse strains and transgenic mice

BALB/cByJ JAX and C57Bl6 females were purchased from Charles Rivers (L’arbresle, France). *K5Cre* transgenic mice, expressing the Cre recombinase under the control of *Krt5* promoter, and *Trp53*^*F/F*^ mice were kindly provided by Dr. J. Jorcano and J. Jonkers, respectively. The adult virgin females used in the experiments were 4 to 6 months old. Age-matched K5Cre;*Trp53*^*F/F*^ females and their control *Trp53*^*F/F*^ littermates were used, as previously described [23]. The care and use of animals were conducted in accordance with the European and National Regulation for the Protection of Vertebrate Animals used for experimental and other scientific purposes (facility license C750517/18). All experimental procedures were ethically approved (ethical approval 02265.02).

### Mammary epithelial cell isolation

Single cells were prepared from a pool of thoracic and inguinal mammary glands harvested from at least three adult virgin mice, as described in detail elsewhere [8]. Briefly, minced tissues were transferred to a digestion solution containing 3 mg/mL collagenase (Roche), 100 units/mL hyaluronidase (Sigma-Aldrich) in CO_2_-independent medium (Gibco Life Technologies) completed with 5% fetal bovine serum (FBS, Lonza), and 2 mM l-glutamine (Sigma-Aldrich) and incubated for 90 min at 37 °C with shaking. Pellets of digested samples were centrifuged and successively treated at 37 °C with solutions of 0.25% trypsin (Life Technologies)/0.1% versen (Biochrom) for 1 min and 5 mg/ml dispase II (Roche)/0.1 mg/mL DNAseI (Sigma-Aldrich) for 5 min. Pellets were treated with a cold ammonium chloride solution (Stem Cell Technologies) and filtered through a nylon mesh cell strainer with 40 mm pores (Fisher Scientific) before immunolabeling.

### Flow cytometry

Freshly isolated mammary cells were incubated at 4 °C for 20 min with the following antibodies: anti-CD45-APC (clone 30-F11; #103112; 1:100), anti-CD31-APC (clone MEC13.3; #102510; 1:100), anti-CD24-BViolet421 (clone M1/69; #101826; 1:50), and anti-CD54-PE (ICAM-1; clone YN1/1.7.4; #116107; 1:50). All antibodies were from BioLegend. Labeled cells were analyzed and sorted out using either a FACSVantage flow cytometer (BD Biosciences) or a MoFlo Astrios cell sorter (Beckman Coulter). Data were analyzed using FlowJo software. Sorted cell population purity was at least 97%.

### Colony- and mammosphere-formation assays

For colony-formation assays, isolated luminal cells were plated on irradiated 3T3 cell feeders in 24-well plates at a density of 500 cells per well and cultured in DMEM/F12 medium supplemented with 10% FBS, 5 μg/mL insulin (Sigma-Aldrich), 10 ng/mL EGF (Invitrogen, Life Technologies), and 100 ng/ml cholera toxin (ICN Biochemicals) for 7–8 days, as previously described [8, 23].

For mammosphere-formation assays, 5000 isolated luminal cells were seeded on ultralow-adherence 24-well plates (Corning) in DMEM/F12 medium supplemented with 2% B27 (Stem Cell Technologies), 20 ng/mL EGF, 20 ng/mL bFGF (GIBCO, Life Technologies), 4 μg/mL heparin (Sigma-Aldrich), 10 μg/mL insulin, and 2% growth-factor-reduced Matrigel (BD Biosciences) for 10–12 days. For second-generation sphere assays, mammospheres were dissociated with 0.05% trypsin (Gibco, Life technologies) and reseeded as described above. When specified, cells were treated with 25–50 ng/ml recombinant mouse HGF (R&D Systems Europe) every 2 days, as described elsewhere [8]. ImageJ software (NIH) was used to count colonies and mammospheres and quantify their size in pixels.

### Immunofluorescence staining and BrdU incorporation assay

The following primary antibodies were used: anti-BrdU (Serotec MCA2060), anti-K5, and anti-K8 (BioLegend; 905501 and 904801). Alexafluor-488 or Alexafluor-594 conjugated secondary antibodies were from Molecular Probes (Invitrogen). Samples were mounted in Prolong Gold antifade reagent with DAPI (Invitrogen, Life Technologies). Image acquisition was performed using a Leica DM 6000B microscope (Wetzlar, Germany) and MetaMorph software (MDS Analytical Technologies Inc.).

Mammospheres resuspended in 50 μL Matrigel were fixed in a 1/3/6 mixture of acetic acid/chloroform/methanol and embedded in paraffin. Sections (7 μm) were cut and dewaxed for immunolabeling, as described [8].

Colonies were incubated with 5 μM BrdU (Sigma) at 37 °C for 1 h and fixed with cold 70% ethanol. Prior to immunolabeling, cells were treated with 2N HCl for 20 min at room temperature and next, with 0.1M borax buffer, as described [23].

### RNA purification and reverse transcription-polymerase chain reaction

Total RNA was extracted from freshly isolated cell populations or dissociated mammospheres using the RNAeasy Microkit (Qiagen). Purified RNA was reverse-transcribed using MMLV H(−) Point reverse transcriptase (Promega), and qPCR was performed by monitoring, in real time, the increase in fluorescence of the QuantiNova SYBR Green PCR Kit (Qiagen) on a LightCycler 480 Real-Time PCR System (Roche). The values obtained were normalized to *Gapdh* levels.

The primers used for qPCR analysis were purchased from SABiosciences/Qiagen or designed using Oligo 6.8 software and synthesized by Eurogentec. The designed primers (for *Trp53*, *Krt5*, *Krt18*, *Elf5*, *Hey1*, *Snai2*, *Trp63*) have been previously documented [8, 23].

### Microarray analysis

Gene expression analysis was performed with total RNA extracted from seven distinct pools of Lu-pos and Lu-neg cells isolated from mammary glands of BALB/cByJ JAX and C57Bl/6 females. Quality control was performed using the Agilent Bioanalyzer and RNA 6000 Pico total RNA Kit (Agilent). The WT-Ovation™ Pico RNA Amplification System (Nugen) was applied from 1 ng of total RNA to generate sufficient amount of biotinylated cDNA. Samples were hybridized on Affymetrix GeneChip Mouse Genome 2.1ST arrays.

Analyses were made using EASANA® (GenoSplice, www.genosplice.com), which is based on the GenoSplice’s FAST DB® release 2014_2 annotations [33]. Data were normalized using quantile normalization and a paired Student’s *t* test was used to compare gene intensities in the different samples. Genes were considered significantly regulated when fold-change between the compared groups was ≥ 1.5 and uncorrected *p* value ≤ 0.05. The molecular and functional interactions of the genes identified were analyzed with KEGG approach (https://www.genome.jp/kegg/).

### Statistical analysis of the data

*p* values were determined using Student *t* test with two-tailed distribution and Welch’s correction, assuming both populations have unequal variance.

## Results and discussion

### Molecular profiles of the luminal subsets separated by ICAM-1

We recently identified ICAM-1 as a robust surface marker allowing the separation of clonogenic luminal progenitors from non-clonogenic ER/PR+ luminal cells in the adult mouse mammary epithelium [8]. This cell adhesion molecule is a well-established target of NF-kappa B signaling, a crucial pathway for mammary development and tumorigenesis [34, 35]. Notably, ICAM-1 appeared more efficient in revealing the functional heterogeneity of the luminal cell population than Sca-1, the most commonly used marker to separate luminal subsets [8, 36].

**Fig. 1:**
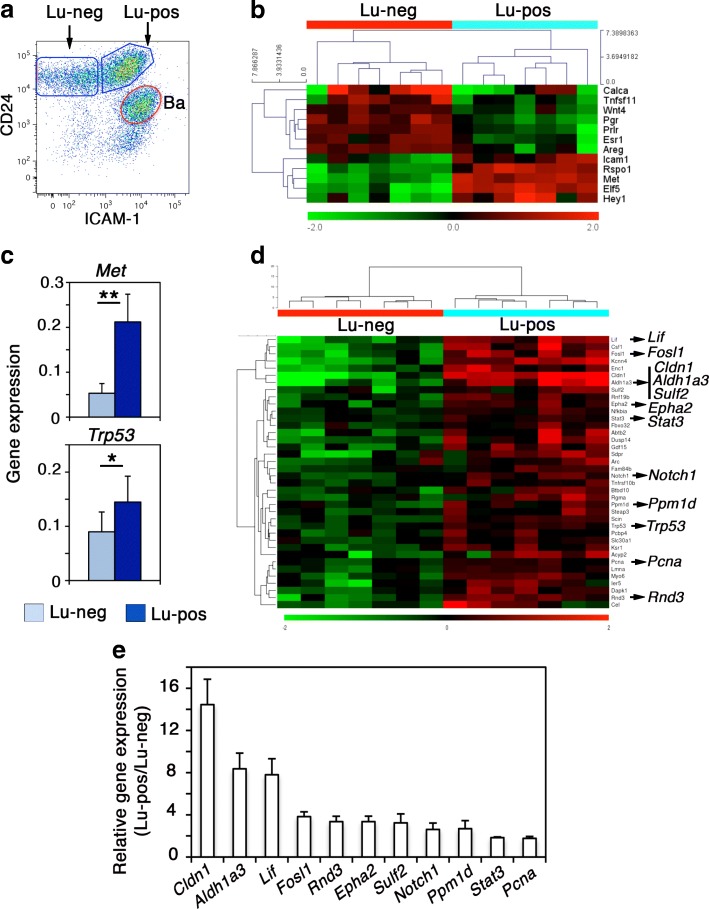
Mammary luminal progenitors express *Met* and *Trp53.* **a** Flow cytometry analysis of CD24 and ICAM-1 expression in mammary cells isolated from adult virgin mice. The gated subsets within the CD24^+^ epithelial cell pool include CD24^+^ ICAM1^+^ basal cells (Ba), CD24^+^ ICAM1^−^ and CD24^+^ ICAM1^+^ luminal cells (Lu-neg and Lu-pos, respectively). **b** Heat map showing expression of the major luminal-specific genes discriminating the hormone-sensing Lu-neg cell population from the clonogenic Lu-pos cell subset separated by ICAM-1. The mean fold-changes in gene expression vary from 1.8 (*Wnt4*) to 6.0 (*Elf5*). **c** Gene expression level of *Met* and *Trp53* in isolated Lu-neg and Lu-pos cells, evaluated by qPCR. Data are the mean ± SEM of 4 separate cell preparations. *p ≤ 0.05, **p ≤ 0.01. **d** Heat map showing the upregulated expression of *Trp53*- and p53-activated target genes in the Lu-pos cell samples. Sixty-eight percent of the genes display a differential expression with a mean fold-change higher than 1.7. **e** Validation of the array data shown in **d** by qPCR analysis. Results are shown as mean ratios (± SEM) between gene expression levels in Lu-pos and Lu-neg cells from at least 3 separate preparations. All the tested genes were significantly overexpressed in Lu-pos compared to Lu-neg cells by at least a factor 2 (*p* ≤ 0.01)

To precisely analyze the molecular characteristics of the luminal cell populations separated by ICAM-1, referred to as Lu-neg and Lu-pos, respectively (Fig. 1a), we compared their gene expression profile using microarrays. Our analysis revealed 2090 modulated genes of which 1115 genes were robustly expressed (log_2_ intensity level ≥ 7). The differential expression of these 1115 genes clustered the two luminal subsets with 447 overexpressed and 668 underexpressed genes in Lu-pos compared to Lu-neg cells (Additional file 1: Figure S1a). As expected from our previously obtained qPCR data [8], the clonogenic Lu-pos cell fraction expressed very low levels of hormone receptors (*Esr1*, *Pgr*, and *Prlr*, encoding ER, PR, and prolactin receptor, respectively) whereas it strongly expressed *Met* and essential regulators of mammary development including *Elf5*, *Hey1*, and *Rspo1* encoding R-spondin1 [37–39] (Fig. 1b). In contrast, the non-clonogenic Lu-neg cells displayed characteristics of hormone-sensing cells, including high expression levels of hormone receptors and local mediators downstream hormonal signaling such as *Wnt4*, *Areg*, *Tnfsf11*, and *Calca*, encoding Wnt4, Amphiregulin, Rankl, and calcitonin, respectively [1] (Fig. 1b). The list of the top 100 modulated genes comprised, in addition to the well-established regulators of the luminal lineage mentioned above, several markers reported to be enriched in ER/PR^−^ luminal progenitors (*Tspan8*, *Aldh1a3*) or hormone-sensing cells (*Ly6a*, *Prom1*) [6, 36, 40] (Additional file 1: Figure S1b).

Kegg pathway analysis indicated that the clonogenic Lu-pos cell fraction displayed numerous upregulated genes associated with the MAPK, PI3K-Akt, NF-kappa B, and Hippo signaling pathways (Additional file 2: Table S1). These pathways are known to play important roles in the control of mammary luminal cell expansion during development and tumorigenesis [15, 16, 35, 41]. Notably, a large set of genes associated with metabolic pathways, glutathione metabolism, and oxidative phosphorylation was downregulated in Lu-pos cells, a hallmark of stem/progenitor cells [42].

### Luminal progenitors identified by ICAM-1 express *Met* and *Trp53* at high level

We previously reported that the ICAM1+ luminal progenitor-enriched population is a target of HGF/Met signaling [8]. Interestingly, our microarray and qPCR data revealed that this cell fraction displayed higher level of *Trp53* than the non-clonogenic ER/PR+ luminal population (Fig. 1c, d). p53 acts mainly as an activator of transcription and, in particular, positively regulates cell cycle arrest, apoptosis, DNA repair, autophagy, and metabolism in a cell- and context-dependent manner [43, 44]. To screen for p53 target genes expressed by the Lu-pos cell fraction, we crossed our data with those from a recent meta-analysis identifying a set of 346 target genes activated by p53 [43]. Of these high-confidence genes, defined as protein-coding genes differentially regulated following p53 activation or inactivation and bound by p53 near the gene locus, a total of 60 genes was found modulated in Lu-pos cells. In addition to *Trp53*, 37 activated p53 target genes were overexpressed in the Lu-pos cell population, revealing a highly significant overlap (*p* = 4.52 × 10^−3^) between these gene sets (Additional file 1: Figure S1c). These included genes encoding signaling receptors (*Epha2*, *Notch1*, *Dapk1*, *Rgma*, *Tnfrsf10b*), cell adhesion and cytoskeleton-associated molecules (*Cldn1*, *Rnd3*, *Myo6*, *Scin*, *Arc*), and enzymes (*Aldh1a3*, *Rnf19b*, *Sulf2*, *Cel*, *Steap3*) and comprised multiple regulators of cell proliferation and differentiation (*Epha2*, *Notch1*, *Lif*, *Csf1*, *Fosl1*), apoptosis (*Tnfrsf10, Dapk1*, *Rgma*, *Stat3*, *Ier5*, *Ppm1d*, *Enc1*), and DNA repair (*Lmna*, *Pcna*) (Fig. 1d). Microarray data were validated on a large set of genes by qPCR (Fig. 1d, e), confirming that in addition to *Trp53* itself, the luminal progenitor-enriched population identified by ICAM-1 overexpressed numerous genes potentially activated by p53.

### Loss of p53 induces the expansion of luminal progenitors without affecting their identity

To study the impact of p53 loss on luminal progenitor characteristics, we used K5Cre;*Trp53*^*F/F*^ mutant mice that were previously characterized in our group [23]. In this model, *Trp53* deletion is targeted in embryonic K5-expressing mammary stem cells, leading to a complete loss of *Trp53* in both luminal and basal cell compartments from the adult mammary epithelium. The functional analysis of the p53-deficient luminal compartment revealed an increased ability to form mammospheres upon serial passages, indicative of a high content in self-renewing luminal stem/progenitor cells.

**Fig. 2:**
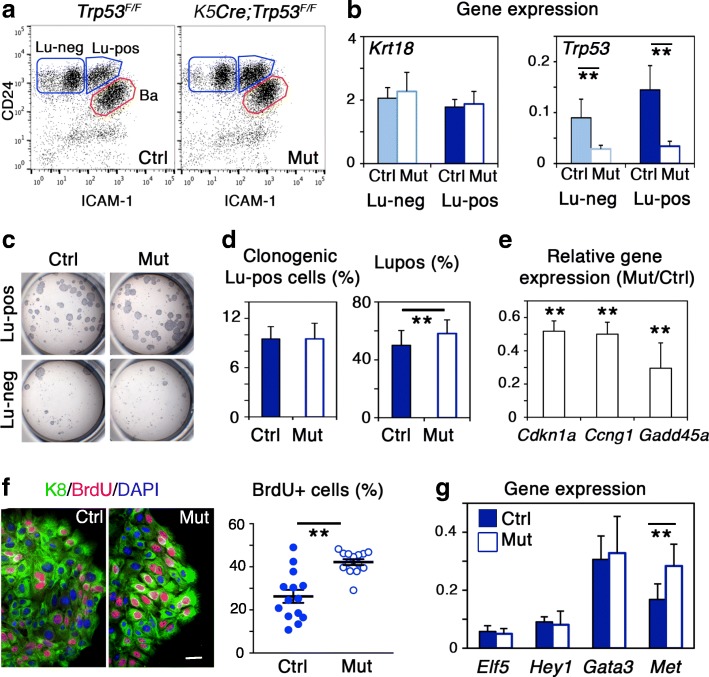
Loss of p53 induces expansion of luminal progenitors overexpressing *Met.* **a** Flow cytometry analysis of CD24 and ICAM-1 expression in mammary cells isolated from *Trp53*^*F/F*^ (Ctrl, control) and *K5Cre*;*Trp53*^*F/F*^ (Mut, mutant) adult virgin mice. **b** qPCR analysis of *Krt18* and *Trp53* expression in Lu-neg and Lu-pos cells isolated from control and mutant adult virgin mice. Data are the mean ± SEM of 4 separate cell preparations. **p ≤ 0.01. **c** Microphotographs of the colonies formed by 500 control and mutant Lu-neg and Lu-pos cells. **d** Left: Percentages of clonogenic cells in control and mutant Lu-pos cell populations. Data are the mean ± SEM of 3 distinct assays. Right: Percentages of Lu-pos cells in control and mutant luminal cell populations calculated from flow cytometry data. Values shown represent the mean ± SEM of 7 separate analyses of distinct cell preparations. **e** Expression of *Cdkn1a*, *Ccng1*, and *Gadd45a* (encoding p21, cyclin G1, and Gadd45a, respectively) in Lu-pos cells isolated from control and mutant adult virgin mice, evaluated by qPCR. Data are shown as mean ratios (± SEM) between gene expression levels in mutant and control Lu-pos cells from 4 separate preparations. **p ≤ 0.01. **f** Left: K8 and BrdU immunodetection in colonies derived from control and mutant Lu-pos cells. DAPI-stained nuclei appear in blue. Bar, 20 μm. Right: Percentages of BrdU^+^ cells. Each point represents counting of one microscope field comprising 100–300 DAPI-stained nuclei. Data are shown as mean ± SEM of countings from 2 distinct experiments. **p ≤ 0.01. **g** Expression levels of the luminal-specific regulators, *Elf5*, *Hey1*, *Gata3*, and *Met* in control and mutant Lu-pos cells, evaluated by qPCR. Data are the mean ± SEM of 3–4 separate preparations. **p ≤ 0.01

To study their molecular characteristics, we purified Lu-neg and Lu-pos cell subsets from mammary cells isolated from K5Cre;*Trp53*^*F/F*^ adult virgin mice and their control littermates, using flow cytometry (Fig. 2a). In line with our previous studies [8, 23], *Trp53* depletion was observed in both mutant luminal cell subsets (Fig. 2b) and the clonogenic activity was concentrated in the Lu-pos cell fraction, in mutant as in control epithelium (Fig. 2c). We found similar clonogenic cell contents in mutant and control Lu-pos populations; however, the proportion of Lu-pos cells within the total luminal compartment was higher in the mutant epithelium (Fig. 2d). These data indicated an expansion of progenitor cells within the luminal cell compartment of K5Cre;*Trp53*^*F/F*^ mutant mice, potentially due to an increased proliferative potential. Consistently, several important p53 target genes encoding negative regulators of cell cycle progression, i.e., p21, cyclin G1, and Gadd45a [43, 44] were repressed in p53-deficient Lu-pos cells (Fig. 2e). In addition, as shown by BrdU incorporation assays, p53-deficient colony-forming cells proliferated more than their control counterparts, generating larger colonies (Fig. 2f).

We did not find any alteration in the expression of *Mdm2* (Additional file 1: Figure S1d), one of the main p53-target genes involved in the complex feedback mechanisms regulating p53 activity [44]. Nonetheless, other important p53 targets potentially involved in feedback regulation and control of apoptosis, such as *Ppm1d*, *Stat3*, and *Tp53inp1* [43], were found underexpressed in mutant luminal progenitor cells (Additional file 1: Figure S1d). Of note, *Pcna* expression also was repressed in the absence of p53 (Additional file 1: Figure S1d). This observation is consistent with data reporting transcriptional activation of PCNA promoter by P53 and suggests a role for Pcna in DNA repair downstream of p53 [43]. Thus, in addition to its stimulatory effect on cell growth, loss of p53 might affect a broad range of luminal progenitor responses.

To further analyze the impact of p53 loss on the molecular characteristics of luminal progenitor cells, we compared expression of several lineage-specific genes in control and mutant Lu-pos cells, using qPCR. Expression levels of the luminal-specific keratin, *Krt18*, and the major transcription factors regulating the luminal progenitor cell fate, *Elf5*, *Hey1*, and *Gata3* [37, 39, 45], were similar in p53-deficient and p53-proficient Lu-pos cells (Fig. 2b, g). Very low levels of the basal-specific keratin, *Krt5*, were detected in mutant as in control Lu-pos cell subsets (Additional file 3: Figure S2a).

Altogether, these results indicate that loss of p53 in the mammary epithelium induced the in vivo expansion of luminal progenitor cells and stimulated their proliferative capacities without altering expression of their major lineage-specific determinants. Accordingly, a recent study reported that p53 loss targeted by the *Krt8* promoter caused the in vivo expansion of mammary luminal cells, increasing their proliferation and cell cycle activity without affecting their luminal fate, until tumor onset at 6–7 months of age [32]*.*

### Loss of p53 induces overexpression of Met signaling components in the mammary epithelium

It has been reported that p53 can regulate Met expression and affect Met signaling via various molecular mechanisms acting at transcriptional and post-transcriptional levels [46]. Interestingly, we found that *Met* expression was 1.7 times higher in mutant than in control Lu-pos cells (Fig. 2g), indicating that p53 loss in luminal progenitors was accompanied by increased Met transcript levels. Similarly, p53 inactivation in ovarian epithelial cells was shown to induce overexpression of *Met* transcripts [29]. This led to elevated levels of phosphorylated Met and to increased cell motility and invasion, even in the absence of HGF stimulation. Loss of p53 function has also been reported to enhance Met signaling in other types of epithelial cells, in particular by promoting Met recycling [30].

In the mammary epithelium, basal cells rather than luminal cells express *Hgf*, suggesting that they might be a source of active HGF and thereby control luminal progenitor function in a paracrine manner [8]. Loss of p53 did not lead to atypical *Hgf* expression in luminal cells; however, higher levels of HGF transcripts were detected in mutant basal cells compared to controls (Additional file 3: Figure S2b).

Collectively, our data suggest that overexpression of *Met* in luminal progenitors and *Hgf* in basal cells might both contribute to the enhanced mammary luminal cell growth observed in K5Cre;*Trp53*^*F/F*^ mutant mice.

### p53-deficient luminal progenitors are stimulated by Met activation but fail to acquire basal cell markers

To investigate the impact of p53 loss on the luminal progenitor response to Met activation, control and mutant luminal Lu-pos cells were grown as mammospheres in the presence or absence of HGF, for 10–12 days, as reported previously [8]. Part of the first generation mammospheres (MS1) was dissociated and replated to obtain second-generation mammospheres (MS2), a strategy used to evaluate the long-term developmental potential of stem/progenitor cells [23].

**Fig. 3:**
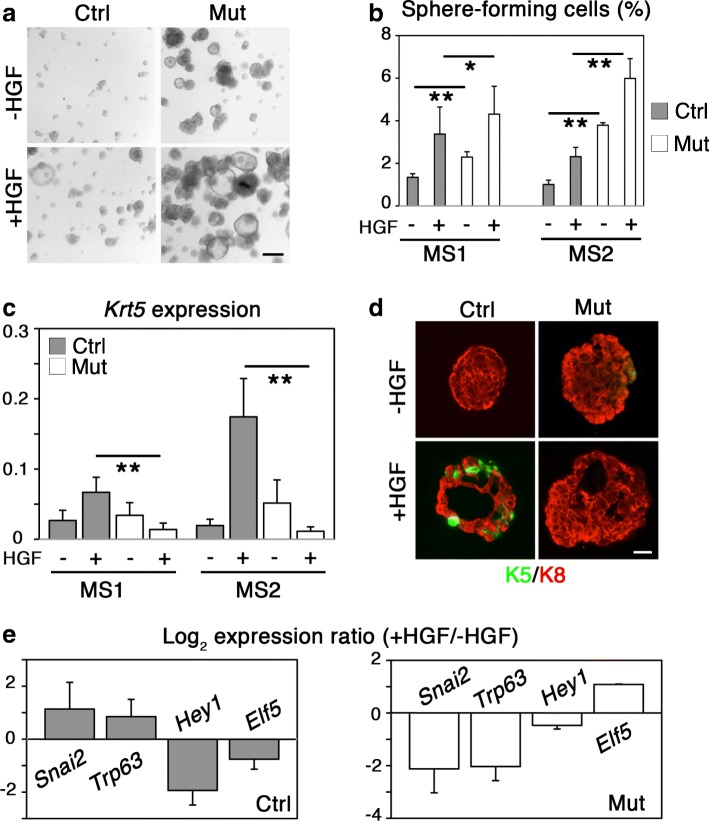
p53-deficient luminal progenitors are stimulated by HGF but fail to acquire basal-specific markers. **a** Microphotographs of secondary mammospheres derived from control and mutant Lu-pos cells cultured in the absence of presence of HGF for 10 days. Bar, 200 μm. **b** Percentages of sphere-forming cells after consecutive passages of 5000 control and mutant Lu-pos cells grown in the absence or presence of HGF. MS1 and MS2 refer to primary and secondary mammospheres. Data are the mean ± SEM of 4 distinct assays. *p ≤ 0.05, **p ≤ 0.01. **c** qPCR analysis of *Krt5* expression in MS1 and MS2 spheres derived from control and mutant Lu-pos cells cultured in the absence or presence of HGF. Data are the mean ± SEM of 4 separate preparations. **p ≤ 0.01. **d** Double K5/K8 staining of sections through secondary spheres derived from control and mutant Lu-pos cells cultured in the absence or presence of HGF. Bar, 40 μm. **e** Comparative expression levels of lineage-specific genes in MS2 spheres derived from control and mutant Lu-pos cells untreated or treated with HGF. qPCR data are expressed as log_2_ ratios of values normalized to *Gapdh.* Comparator values were those obtained with untreated cells. Data are the mean ± SEM of 3 distinct preparations

In line with our previous findings [23], we observed that in the absence of any treatment, p53-deficient Lu-pos cells formed more and larger MS1 and MS2 spheres than control cells (Fig. 3a, b, Additional file 3: Figure S2c). Mutant Lu-pos cells responded to HGF stimulation by an increase in the number and size of MS1 and MS2 spheres (Fig. 3a, b, Additional file 3: Figure S2c), showing that Met activation stimulated the clonogenic activity of p53-deficient luminal progenitors, as previously found for p53-proficient cells [8]. Of note, MS1 control spheres displayed a reduced level of *Trp53* upon HGF stimulation (Additional file 3: Figure S2d). This suggests that Met activation might negatively regulate p53 signaling in luminal progenitors, a mechanism that could account for their increased developmental potential upon HGF stimulation. In line with this hypothesis, Met signaling has been reported to inhibit p53 activity via Mdm2 in hepatocytes, promoting their survival in vivo and in vitro [28].

Luminal progenitors stimulated by HGF acquire basal cell markers [8]. We therefore examined if loss of p53 affected K5 expression in HGF-treated MS1 and MS2 spheres. Using qPCR, we found that unlike control, p53-deficient luminal progenitors failed to upregulate *Krt5* upon Met activation (Fig. 3c). Immunodetection studies confirmed that control MS2 spheres contained numerous K5-expressing cells, whereas p53-deficient spheres were almost exclusively composed of K8^+^ luminal cells (Fig. 3d).

Several antagonistic regulators of the balance between mammary luminal and basal phenotypes have been identified. Notch signaling that specifies the luminal cell fate negatively regulates the expression of ΔNp63, a p63 isoform required for the maintenance of basal cell characteristics [37, 47]. Elf5 promotes luminal cell differentiation by transcriptionally repressing Slug/Snail2, an EMT-associated transcription factor crucial for the mammary basal cell identity [48, 49]. As p53-deficient luminal progenitors failed to acquire K5 upon Met activation, we analyzed the expression of *Hey1* (a major effector of Notch signaling), *Trp63*, *Elf5*, and *Snai2* in control and mutant luminal progenitors stimulated by HGF. As expected, the absence of K5^+^ cells in HGF-stimulated mutant spheres was accompanied by low levels of *Trp63* and *Snai2* expression, whereas these basal-specific transcription factors were strongly induced in HGF-treated control spheres containing K5^+^ basal cells (Fig. 3e). Consistently, the luminal-specific regulator *Elf5*, repressed in HGF-treated control spheres, was upregulated in mutant cells upon HGF treatment, whereas *Hey1*, strongly downregulated in HGF-stimulated control spheres, was not significantly modulated in HGF-treated mutant spheres (Fig. 3e).

**Fig. 4:**
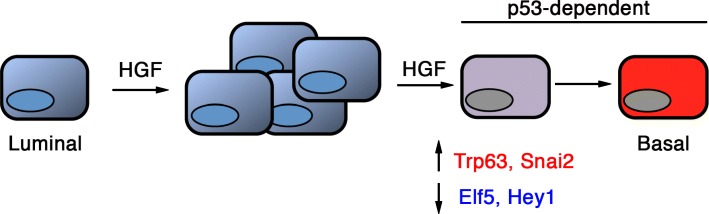
p53 controls the plasticity of mammary luminal progenitor cells downstream of Met signaling. HGF stimulates luminal progenitor activity and favors acquisition of basal-specific markers. The luminal-to-basal switch induced by Met activation depends on p53 function

Collectively, these results show that p53 function is required for luminal progenitors to acquire basal characteristics upon Met activation (Fig. 4). Of note, in basal cells, loss of p53 did not affect the expression of *Trp63* and *Snai2* (Additional file 3: Figure S2e), two genes potentially regulated by p53 signaling [50–52]. This indicates that a p53-deficient context is compatible with expression of mammary basal cell characteristics.

## Conclusions

Our study provides a molecular analysis of the two main subsets of the mammary luminal compartments, luminal progenitors and hormone-sensing luminal cells. It reveals that luminal progenitors, the cellular targets of HGF/Met signaling in the mammary epithelium, overexpress p53 and numerous activated p53 target genes.

A growing body of evidence indicates that p53 can regulate self-renewal, differentiation, and plasticity of embryonic and adult stem/progenitor cells [21, 22]. Accordingly, we report that in vivo, loss of p53 induced the expansion of luminal progenitors and stimulated their proliferative and self-renewal activities. However, it did not affect their luminal identity. Moreover, p53-deficient luminal progenitors stimulated by HGF ex vivo failed to acquire basal-specific features, showing that p53 controls the plastic behavior of luminal progenitors downstream of Met activation. In the mammary epithelium, loss of p53 has been reported to promote self-renewal and symmetric division of stem cells [53, 54]. This mechanism might account, at least partly, for the inability of p53-deficient luminal progenitors to undergo a luminal-to-basal switch upon Met activation.

Interestingly, we found *Met* upregulated in p53-deficient luminal progenitors and *Trp53* repressed in HGF-stimulated luminal progenitors. This suggests a bi-directional crosstalk between Met- and p53-mediated signaling pathways in the control of luminal progenitor function and illustrates the cooperative molecular interplay between oncogene activation and tumor suppressor inactivation in the mammary epithelium.

Basal-type TNBCs are thought to originate from the aberrant amplification of luminal progenitors acquiring certain basal cell features [12–16]. Loss of P53 function is considered as an early key event in TNBC development leading to multiple genomic lesions and molecular alterations [17, 27]. Those include *MET* amplification, *MET* mutation, and MET signaling hyperactivation [18, 26]. Recent studies, using p53-deficient mouse models, suggest that Met activation synergizes with p53 loss to induce triple-negative mammary tumors, with basal or claudin-low characteristics [31, 32]. Our data indicate that neither p53 loss alone nor p53 loss combined with Met signaling activation caused an early detectable cell fate alteration in luminal progenitors. This process might occur at long-term during mammary tumorigenesis, as a consequence of the complex molecular abnormalities triggered by p53 loss.

## Additional files

Additional file 1: **Figure S1.** Molecular characteristics of the luminal subsets isolated from control and *K5Cre*;*Trp53*^*F/F*^ adult virgin mice using ICAM-1. (a) Heat map showing hierarchical clustering of the 7 Lu-pos and Lu-neg cell samples analyzed. A total of 1115 genes with a minimal intensity level of 7 displayed a fold-change ≥ 1.5. (b) Expression heat map of the top 100 modulated genes. (c) Venn diagram showing the number of activated p53 target genes differentially expressed in Lu-pos and Lu-neg cell populations. (d) Relative expression levels of p53 target genes in Lu-pos cells isolated from control and *K5Cre*;*Trp53*^*F/F*^ mutant adult virgin mice. The qPCR data are shown as mean ratios ± SEM between gene expression levels in mutant and control Lu-pos cells from at least 3 separate preparations. *p ≤ 0.05, **p ≤ 0.01. (PDF 8195 kb)

Additional file 2: **Table S1.** KEGG pathway analysis on genes differentially expressed in Lu-pos compared to Lu-neg cell populations. (XLSX 12 kb)

Additional file 3: **Figure S2.** Characteristics of the basal and luminal populations isolated from control and *K5Cre*;*Trp53*^*F/F*^ adult virgin mice, and of mammospheres derived from control and p53-deficient luminal progenitors. (a) Expression levels of the luminal- and basal-specific keratins (*Krt18* and *Krt5*) in control and p53-deficient Lu-pos cells, evaluated by qPCR. Data are the mean ± SEM of 4 separate preparations. (b) *Hgf* expression in control and p53-deficient Lu-neg, Lu-pos, and basal cell populations. The qPCR data are shown as mean ± SEM of 3 separate preparations. *p ≤ 0.05. (c) Size distribution of MS2 spheres generated by control and p53-deficient Lu-pos cells grown with or without HGF. Sphere areas were estimated in pixels on phase contrast pictures, using ImageJ software. Spheres of ≤ 400 and > 400 pixels were defined as small and large, respectively. Data are the mean ± SEM of 4 separate preparations. (d) *Trp53* expression in MS1 and MS2 spheres derived from control and p53-deficient Lu-pos cells grown with or without HGF. The qPCR data are shown as mean ± SEM of 4 separate preparations. *p ≤ 0.05. (e) Expression levels of *Trp53*, *Trp63*, and *Snai2* in basal cells isolated from control and *K5Cre*;*Trp53*^*F/F*^ mutant adult virgin mice, evaluated by qPCR. Data are the mean ± SEM of 3 separate preparations. **p ≤ 0.01. (PDF 5273 kb)

## Abbreviations

BrdU: 5-Bromo-2′-deoxyuridine
EMT: Epithelial-mesenchymal transition
ER: Estrogen receptor
FBS: Fetal bovine serum
HGF: Hepatocyte growth factor
PR: Progesterone receptor
Prlr: Prolactin receptor
TNBC: Triple-negative breast cancer
