# Supplementary figures and images for: p53 controls the plasticity of mammary luminal progenitor cells downstream of Met signaling
Source: Breast Cancer Res. 2019 Jan 25;21:13. doi: 10.1186/s13058-019-1101-8 (PMC6346556; doi:10.1186/s13058-019-1101-8)

Figure S1

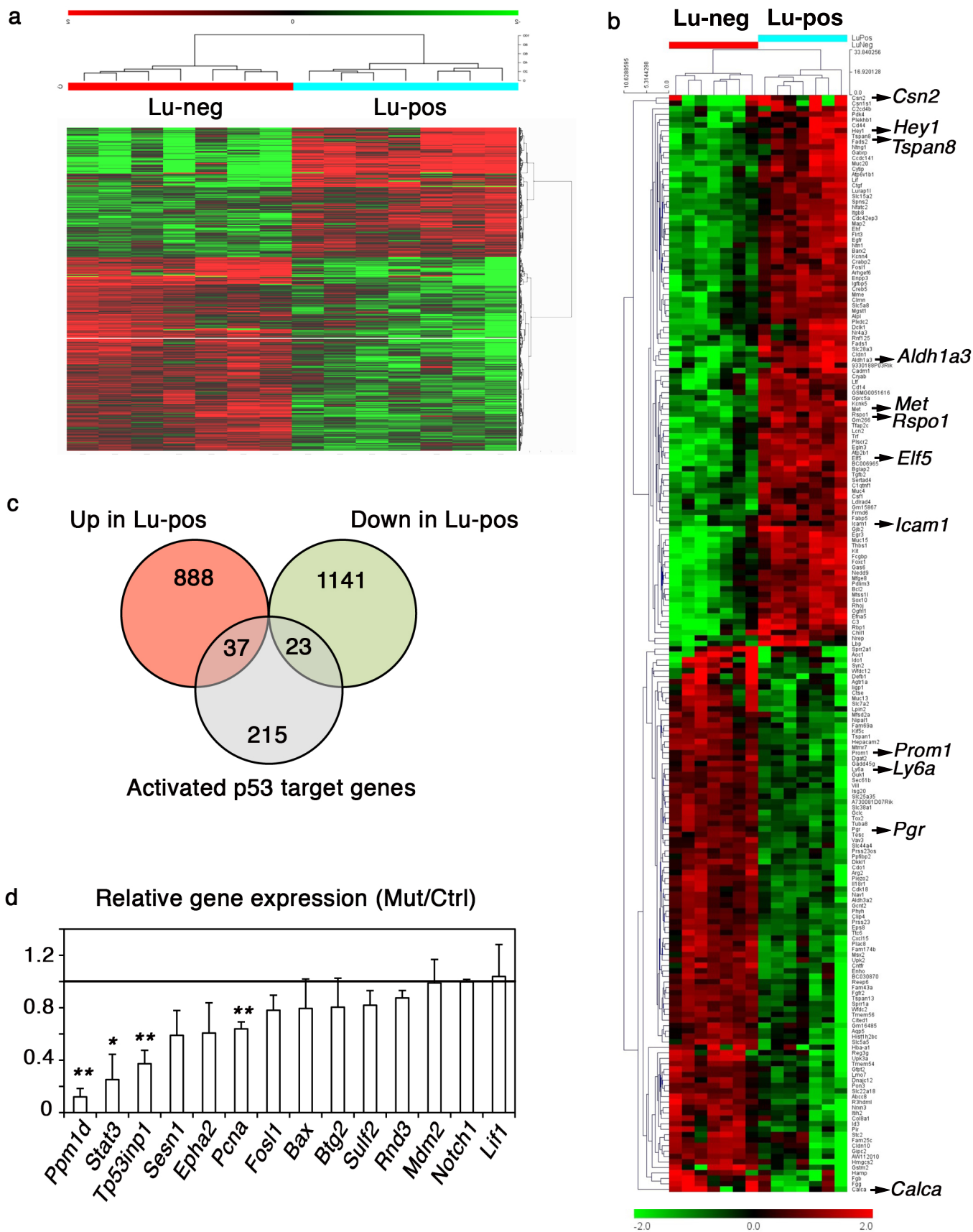

Supplement: Supplementary file 1 — Figure S1. Molecular characteristics of the luminal subsets isolated from control and K5Cre;Trp53F/F adult virgin mice using ICAM-1. (a) Heat map showing hierarchical clustering of the 7 Lu-pos and Lu-neg cell samples analyzed. A total of 1115 genes with a minimal intensity level of 7 displayed a fold-change ≥ 1.5. (b) Expression heat map of the top 100 modulated genes. (c) Venn diagram showing the number of activated p53 target genes differentially expressed in Lu-pos and Lu-neg cell populations. (d) Relative expression levels of p53 target genes in Lu-pos cells isolated from control and K5Cre;Trp53F/F mutant adult virgin mice. The qPCR data are shown as mean ratios ± SEM between gene expression levels in mutant and control Lu-pos cells from at least 3 separate preparations. *p ≤ 0.05, **p ≤ 0.01. (PDF 8195 kb) [file 13058_2019_1101_MOESM1_ESM.pdf]

**Figure S2**

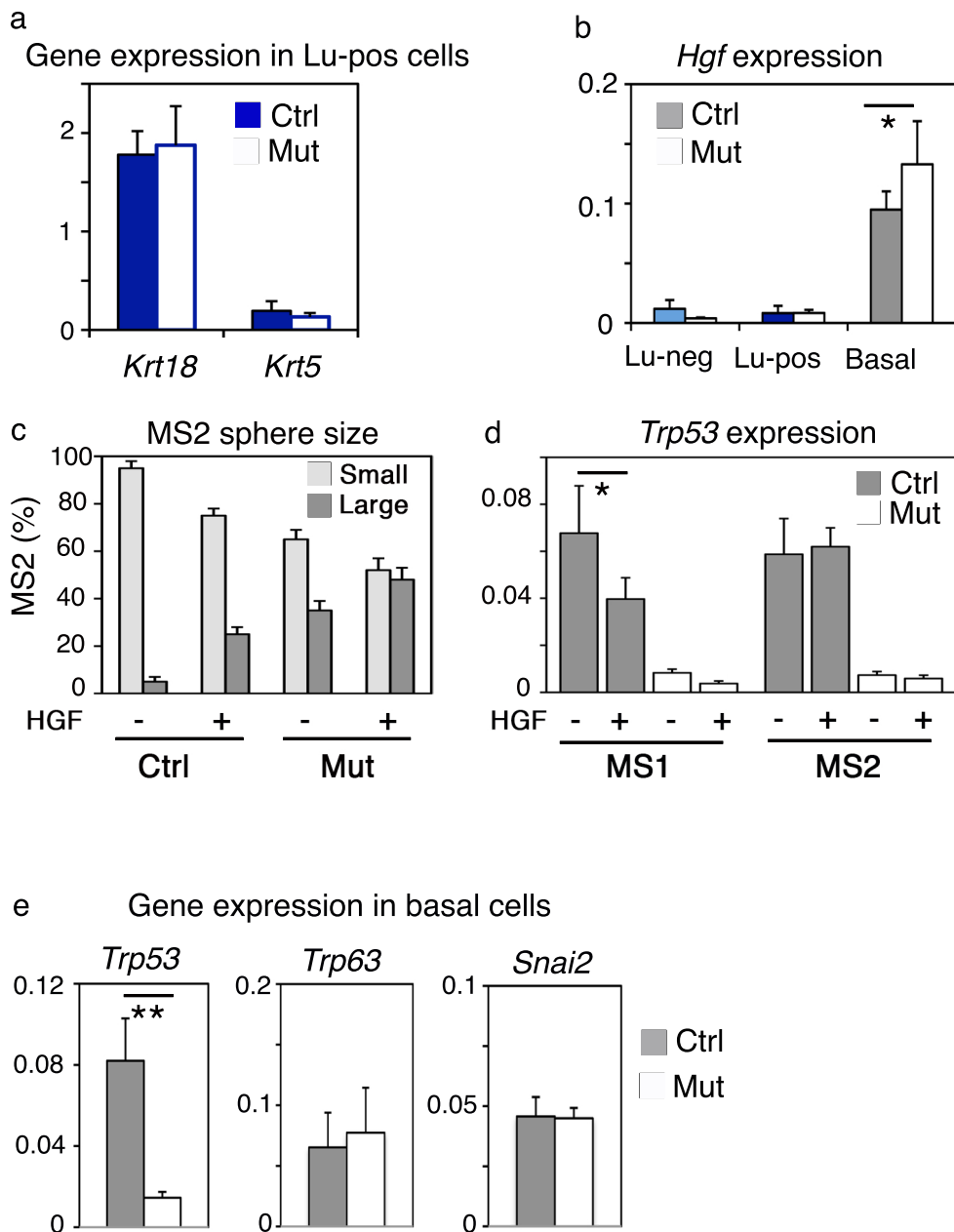

Supplement: Supplementary file 3 — Figure S2. Characteristics of the basal and luminal populations isolated from control and K5Cre;Trp53F/F adult virgin mice, and of mammospheres derived from control and p53-deficient luminal progenitors. (a) Expression levels of the luminal- and basal-specific keratins (Krt18 and Krt5) in control and p53-deficient Lu-pos cells, evaluated by qPCR. Data are the mean ± SEM of 4 separate preparations. (b) Hgf expression in control and p53-deficient Lu-neg, Lu-pos, and basal cell populations. The qPCR data are shown as mean ± SEM of 3 separate preparations. *p ≤ 0.05. (c) Size distribution of MS2 spheres generated by control and p53-deficient Lu-pos cells grown with or without HGF. Sphere areas were estimated in pixels on phase contrast pictures, using ImageJ software. Spheres of ≤ 400 and > 400 pixels were defined as small and large, respectively. Data are the mean ± SEM of 4 separate preparations. (d) Trp53 expression in MS1 and MS2 spheres derived from control and p53-deficient Lu-pos cells grown with or without HGF. The qPCR data are shown as mean ± SEM of 4 separate preparations. *p ≤ 0.05. (e) Expression levels of Trp53, Trp63, and Snai2 in basal cells isolated from control and K5Cre;Trp53F/F mutant adult virgin mice, evaluated by qPCR. Data are the mean ± SEM of 3 separate preparations. **p ≤ 0.01. (PDF 5273 kb) [file 13058_2019_1101_MOESM3_ESM.pdf]
